# Complete Genome Sequence of Human Adenovirus 7 Associated with Fatal Adult Pneumonia

**DOI:** 10.1128/genomeA.01204-16

**Published:** 2016-10-27

**Authors:** Svetlana B. Yatsyshina, Margarita R. Ageeva, Andrey A. Deviatkin, Ekaterina V. Pimkina, Mikhail L. Markelov, Vladimir G. Dedkov, Marina V. Safonova, Elena Y. Shumilina, Alexander N. Lukashev, German A. Shipulin

**Affiliations:** a The Central Research Institute for Epidemiology of Rospotrebnadzor, Moscow, Russia; b Chumakov Institute of Poliomyelitis and Viral Encephalitides, Moscow, Russia

## Abstract

Human adenovirus 7 (hAdv7) 19BOVLB/Volgograd/Rus/2014 was isolated from the autopsy material from an adult with fatal pneumonia in Volgograd, Russia, in March 2014. Whole-genome sequencing of the virus isolate was performed.

## GENOME ANNOUNCEMENT

Human adenoviruses are divided into seven species (A to G) and about 60 types. They can affect different organs and tissues, causing a wide range of clinical manifestations. Respiratory infections are associated with species B, C, and E. Severe respiratory adenoviral infections are usually associated with adenoviruses species B, serotypes 7 and 3 (1).

The patient, a 49-year-old woman with concomitant coronary atherosclerosis, was diagnosed a bilateral subtotal pneumonia complicated by adult respiratory distress syndrome. Clinical manifestations included cough, fever, and headache. On the ninth day of the disease, the condition of the patient broke down significantly. Despite antibacterial, antiviral, anticoagulant therapy, and breathing support, the patient died on the 10th day of illness.

Autopsy samples were PCR positive for adenovirus and negative for other respiratory pathogens. Adenovirus was isolated from the lung of the patient on HeLa cell culture. A genome library was indexed by PCR using Nextera-compatible adaptors. Sequencing was performed using Illumina sequencing-by-synthesis on the MiSeq platform using the 2 × 250-nucleotide (nt) paired-end protocol. Samples were demultiplexed using the Illumina software, and fastq files were generated.

The adenovirus library yielded a total of 160,758 sequence reads. Reads were filtered by quality (Q20); adapter sequences were removed by Trimmomatic-0.32 (2). A *de novo* assembly was generated using SPAdes-3.0 (3). The reads were then remapped to the assembly with Bowtie 2 (4). Alignments were processed using SAMtools (5) and the pysamstats software to estimate genome coverage.

Overall, the assembly generated three contigs (17,179 nt, 15,473 nt, and 2,048 nt). The median coverage depths were 315×, 95×, and 30×, respectively. Minimal coverage depth was 3×. Gaps between contigs were filled by Sanger sequencing, and termini were analyzed by rapid amplification of cDNA ends (RACE).

A complete genome of 19BOVLB/Volgograd/Rus/2014 was 35,214 bp in length, with GC content of 51.1%. It is predicted to contain 48 coding sequences and seven genes. A comparison of the genome with that of other hAdv7 strains revealed homologies of 89.9% to 99.9%. 19BOVLB/Volgograd/Rus/2014 has the highest homology with strain 0901HZ/ShX/CHN/2009, which is also associated with fatal pneumonia. Genomes differ from each other for four one-nucleotide substitutions and for the 24-nucleotide-long deletion (nt 8450 to 8474) in 19BOVLB/Volgograd/Rus/2014, causing a lack of eight amino acids in each of two proteins translated in different reading frames, a 12.6-kDa early protein, and a preterminal protein (pTP). The function of 12.6-kDa early protein is still unknown. pTP acts as protein primer, and it binds to DNA and adenoviral DNA polymerase. pTP has a tripartite structure, which includes a large noncontiguous surface required for interaction with DNA polymerase, an N-terminal DNA binding domain, and a C-terminal regulatory domain (6). The deletion touches on the C-terminal region and may affect DNA binding.

Using the SWISS-MODEL application (http://swissmodel.expasy.org), structural models of these proteins were built. The most likely model of the hypothetical 12.6-kDa early protein includes small amounts of β-structure interspersed with unstructured regions. The model of pTP includes four α-structure regions. The same models were obtained for strain 0901HZ/ShX/CHN/2009 proteins.

Regardless of possible changes being due to the deletion, this adenovirus strain may evoke severe infection in immunocompetent patient.

### Accession number(s).

The GenBank accession number for hAdv7 strain 19BOVLB/Volgograd/Rus/2014 is KU361344.
